# Die Gewährleistung von Krankheitshilfen bei asylsuchenden Menschen: Zweiklassenmedizin in Deutschland?

**DOI:** 10.1007/s00103-020-03215-7

**Published:** 2020-09-22

**Authors:** Alfons Hollederer

**Affiliations:** Fachbereich 01 Humanwissenschaften, Professur Theorie und Empirie des Gesundheitswesens, Universität Kassel, Arnold-Bode-Str. 10 (WISO C), 34109 Kassel, Deutschland

**Keywords:** Asylsuchende, Geflüchtete, Zugang zur Gesundheitsversorgung, Menschenrechte, Asylum seekers, Refugees, Access to health care, Human rights

## Abstract

**Hintergrund:**

Es gibt im Bereich des Asylbewerberleistungsgesetzes (AsylbLG) zwei Möglichkeiten der Leistungsgewährung mit praktischer Relevanz für die Gesundheitsversorgung (abhängig von der Voraufenthaltszeit): die Grundleistungen und die Leistungen in besonderen Fällen analog zum Sozialgesetzbuch (SGB) XII.

**Methodik:**

Die Sekundärdatenanalyse untersucht das Leistungsgeschehen im Krankheitsfall bei den Leistungsempfängern nach dem AsylbLG beim Forschungsdatenzentrum der Statistischen Ämter des Bundes und der Länder. Dem untersuchten Personenkreis wurde noch keine Flüchtlingseigenschaft bzw. Asylberechtigung zuerkannt.

**Ergebnisse:**

Zum Stichtag 31.12.2018 bezogen 423.201 Personen in Deutschland Leistungen nach dem AsylbLG. Davon war gut ein Drittel Frauen. Das Durchschnittsalter betrug 24 Jahre. Über die Hälfte stammte aus Asien. Über ein Drittel aller Leistungsempfänger befand sich in ambulanter (33,5 %) oder stationärer Behandlung (1,3 %). Zwischen den Bundesländern variierten die Leistungen zur Hilfe bei Krankheit sowie die gesundheitsbezogenen Pro-Kopf-Bruttoausgaben sehr stark. Die Gewährung von Leistungen bei Krankheit war in Aufnahmeeinrichtungen relativ gering. Mit Gesundheitskarte war die Inanspruchnahme stationärer Behandlung generell höher.

Die gesundheitsbezogene Regelversorgung mit Hilfen in besonderen Fällen (§2 AsylbLG analog SGB XII) erreichte einen größeren Anteil an leistungsberechtigten Menschen mit 42,7 % am Jahresende als die Minimalversorgung nach §3 AsylbLG mit 29,0 %. Sie verursachte trotzdem im Vergleich weniger Bruttoausgaben.

**Schlussfolgerung:**

Es wird empfohlen, §2 AsylbLG schon bei einer Voraufenthaltszeit ab 3 Monaten anzuwenden, um frühzeitiger die Hilfen analog Kap. 5–9 SGB XII gewähren zu können. Eine flächendeckende Einführung der Gesundheitskarte würde den Zugang verbessern.

## Abstract (en)

**Purpose:**

In the area of the Asylum Seeker Benefits Act (AsylbLG), there are two options for granting benefits that have practical relevance to health care (depending on the period of previous residence): minimum provision and benefits in special cases, analogous to the Social Code Book XII (SGB XII).

**Method:**

This secondary data analysis examines the performance of benefits in the case of illness among benefit recipients according to the AsylbLG at the Research Data Centre of the Federal and State Statistical Offices. The examined group of individuals have not yet been granted refugee status or asylum entitlement.

**Results:**

As of 31 December 2018, 423,201 persons in Germany were receiving benefits according to the AsylbLG. A good third of these were women. The average age was 24 years, and more than half came from Asia. More than one-third of all benefit recipients were in outpatient (33.5%) or inpatient treatment (1.3%). Among the federal states, benefits for assistance in the case of illness as well as the health-related per capita gross expenditure varied considerably. The provision of illness benefits in reception facilities was relatively low. With the health card, the use of inpatient treatment was higher. The standard health-related provision of assistance in special cases (§2 AsylbLG analogous to SGB XII) reached a higher share of people entitled to benefits at the end of the year (42.7%) than the minimum provision according to §3 AsylbLG (29.0%). Nevertheless, it caused comparatively less gross expenditure.

**Conclusion:**

It is recommended to apply §2 AsylbLG for a prestay period of three months or longer in order to be able to grant assistance analogous to Chapters 5 to 9 SGB XII earlier. Nationwide introduction of an electronic health insurance card for asylum seekers would improve access.

## Einleitung

Menschrechte sind unteilbar! Deutschland hat sich als Vertragsstaat des Internationalen Paktes über wirtschaftliche, soziale und kulturelle Rechte verpflichtet, „das Recht eines jeden auf das für ihn erreichbare Höchstmaß an körperlicher und geistiger Gesundheit“ anzuerkennen und die Voraussetzungen zu schaffen, „die für jedermann im Krankheitsfall den Genuss medizinischer Einrichtungen und ärztlicher Betreuung sicherstellen“ (Art. 12 Abs. 1 und 2). Weitere Konkretisierungen der Zugänglichkeit in die Gesundheitsversorgung finden sich für diskriminierungsgefährdete Gruppen in Art. 25 der UN-Behindertenrechtskonvention (CRPD), in Art. 12 der UN-Frauenrechtskonvention (CEDAW) und in Art. 24 der UN-Kinderrechtskonvention (CRC). So ist sicherzustellen, „dass keinem Kind das Recht auf Zugang zu derartigen Gesundheitsdiensten vorenthalten wird“ (Art. 24 Absatz 1). In Deutschland ist aber bei Flüchtlingen und ihren Familienangehörigen eine starke Diskrepanz zwischen dem Anspruch der universellen Menschenrechte und der (Nicht‑)Gewährung von Krankenhilfen auf Grundlage der Asylgesetze zu konstatieren [1, 2]. Bei der Gesundheitsversorgung von Asylbewerbern werden in Deutschland große Versorgungslücken in vielen Analysen übereinstimmend beobachtet [3–8]. Der Ausschuss für wirtschaftliche, soziale und kulturelle Rechte der Vereinten Nationen drückt seine Besorgnis aus, dass „asylum-seekers’ access to health care is restricted to acute and painful conditions for the first 15 months of their stay in Germany and that their access to health care is further limited …“ [9].

Die Versorgung von Flüchtlingen ist aufgrund der föderalen Struktur in Deutschland durch verschiedene Zuständigkeiten auf der Bundes‑, Länder- und kommunalen Ebene geprägt. Für die Durchführung des Asylverfahrens ist der Bund bzw. das Bundesamt für Migration und Flüchtlinge (BAMF) zuständig. Die Verteilung der ankommenden Flüchtlinge auf die Bundesländer orientiert sich an dem Königsteiner Schlüssel und damit nach Steueraufkommen und Bevölkerungszahlen. Die Länder sind nach dem Asylverfahrensgesetz verpflichtet, für die Unterbringung von Asylsuchenden zu sorgen und die Geld- und Sachleistungen zu ihrer Existenzsicherung zu gewährleisten. Nach der Entlassung aus der Aufnahmeeinrichtung folgt die Verteilung innerhalb des Landes in Gemeinschaftsunterkünfte und dezentrale Unterbringungsformen. Es bestehen erhebliche Unterschiede in der Administration zwischen den Bundesländern und in den Kommunen. Asylbewerber und sonstige ausländische Flüchtlinge, die keinen dauerhaften Aufenthaltsstatus besitzen, erhalten im Falle der Hilfsbedürftigkeit soziale Leistungen nach Maßgabe des vorrangigen Asylbewerberleistungsgesetzes (AsylbLG). Der Kreis der Leistungsberechtigten, die sich tatsächlich im Bundesgebiet aufhalten müssen, ist nach §1 Abs. 1 AsylbLG normiert und mit Voraussetzungen verbunden. Leistungsberechtigt sind Ausländer, die ein Asylgesuch geäußert haben bzw. eine Aufenthaltsgestattung nach dem Asylgesetz, eine Aufenthaltserlaubnis oder eine Duldung nach dem Aufenthaltsgesetz besitzen. Zu den leistungsberechtigten Gruppen zählen auch Personen, die über einen Flughafen einreisen wollen und denen die Einreise noch nicht gestattet ist. Weiterhin sind Personen leistungsberechtigt, die vollziehbar ausreisepflichtig sind oder einen Folgeantrag auf Asyl stellen. Hinzu kommen die Ehegatten, Lebenspartner oder minderjährigen Kinder der leistungsberechtigten Personen, ohne dass sie selbst die Voraussetzungen erfüllen.

Das Zugangsverfahren zu medizinischen Leistungen ist in den Bundesländern verschieden reguliert. Für den Arztbesuch benötigen die Leistungsberechtigungen meist einen Berechtigungs- oder Behandlungsschein, dessen Voraussetzungen zur Ausstellung die zuständige Behörde selbst prüft und entscheidet. Darüber hinaus ist nicht nur der Zugang zur Gesundheitsversorgung, sondern auch die freie Arztwahl eingeschränkt. Außerdem dürfen die Allgemeinmediziner im Krankenscheinverfahren in der Regel nicht zu Fachärzten überweisen. Der Übergang von einzelfallbezogenen Krankenscheinausgaben auf ein modernes Gesundheitskartensystem ist allerdings in einem Großteil der Bundesländer in Kooperation mit der gesetzlichen Krankenversicherung gelungen (Landesrahmenvereinbarungen nach §264 Abs. 1 Sozialgesetzbuch (SGB) V aktuell in Berlin, Brandenburg, Bremen, Hamburg, Niedersachsen, Nordrhein-Westfalen, Rheinland-Pfalz, Schleswig-Holstein und Thüringen). In der Umsetzung der gesetzlichen Vorgaben des AsylbLG werden im Verwaltungsvollzug die Ermessensspielräume unterschiedlich genutzt. Razum et al. [10] drücken es pointiert so aus, dass der Zufall über den Zugang zur Gesundheitsversorgung für Flüchtlinge in Abhängigkeit von den Zuweisungen des Bundeslandes und der Kommune sowie der juristischen Kenntnisse der behandelnden Ärzte entscheidet. Eine Befragung von Gesundheitsämtern in Deutschland ergab, dass die gesundheitliche Versorgung von Asylsuchenden in Deutschland außerordentliches Verbesserungspotenzial aufweist [6]. So fehlten strukturelle Ressourcen für die Koordination der Versorgung in den zuständigen Ämtern. Als verbesserungsbedürftig wurden die Standardisierung in den Abläufen, zügige Durchführung von Impfungen, standardisierte Erhebung und Übermittlung gesundheitsbezogener Informationen sowie Konzentration auf wichtige Infektionskrankheiten erachtet [6]. Es hat sich auch herausgestellt, dass die auslösenden Infektionskrankheiten bei Ausbrüchen in Gemeinschaftsunterkünften für Asylsuchende häufig erst in Deutschland erworben wurden [11].

In Deutschland liegen zur Gesundheitsversorgung von Asylsuchenden und Flüchtlingen nur wenige repräsentative Daten und systematische Gesundheitsberichte vor [7]. Empirischen Arbeiten zur medizinischen Versorgung von Asylsuchenden kommt daher besondere Wichtigkeit zu [12]. Die Sekundärdatenanalyse zielt deshalb darauf, das Leistungsgeschehen im Krankheitsfall bei den Leistungsempfängern nach dem AsylbLG zu explorieren sowie nach regionalen, sozialen und gesundheitssystemischen Unterschiedshypothesen zu analysieren. Die Gewährung von Krankheitshilfen bei asylsuchenden Menschen ist immanent mit den Asylgesetzen und der Sozialgesetzgebung verbunden, die nicht nur das Leistungsrecht, sondern auch die Begriffe und die Bundesstatistiken determinieren. Die vorliegende Arbeit führt die Daten separater Bundesstatistiken zusammen und hat die Analyse verschiedener Gewährleistungsprinzipien in der Gesundheitsversorgung nach Reichweite, Regionen, Bruttoausgaben und Gesundheitskarte zum Ziel. Es werden erstmalig Prävalenzraten der ambulanten und stationären Hilfen bei Krankheit nach dem AsylbLG für Bundesländer und soziodemografische Gruppen berechnet. Im Abschnitt Methode wird zunächst auf die essenziellen sozialrechtlichen Grundlagen und danach auf die Asylbewerberleistungsstatistiken eingegangen. Es schließen sich im Ergebnisteil die Sekundäranalysen zum Leistungsgeschehen im Krankheitsfall bei den Leistungsempfängern nach dem AsylbLG auf Basis der Forschungsdatensätze an. Die Diskussion der empirischen Ergebnisse und ein Fazit runden die Arbeit ab.

## Methode

Die Asylbewerberregelleistungen werden entweder als a) sogenannte Grundleistungen oder b) in besonderen Fällen in Form von laufender Hilfe zum Lebensunterhalt gewährt:

Zu a) Die Grundleistungen nach §3 AsylbLG werden zur Deckung des notwendigen Bedarfs an Ernährung, Unterkunft, Heizung, Kleidung, Gesundheitspflege sowie Gebrauchs- und Verbrauchsgütern des Haushalts gewährt. Sie werden vorrangig als Sachleistung erbracht. Zu den Grundleistungen *können* in einer speziellen Bedarfssituation besondere Leistungen bei Krankheit, Schwangerschaft und Geburt (§4 AsylbLG) und sonstige Leistungen (§6 AsylbLG) genehmigt werden (Stichwort „Minimalversorgung“).

Zu b) In den besonderen Fällen wird nach §2 AsylbLG das Zwölfte Sozialgesetzbuch (SGB XII) auf diejenigen Leistungsberechtigten angewandt, die sich seit 18 Monaten ohne wesentliche Unterbrechung im Bundesgebiet aufhalten und die Dauer des Aufenthalts nicht rechtsmissbräuchlich selbst beeinflusst haben (die vorgeschriebene Voraufenthaltszeit lag in 2018 noch bei 15 Monaten). Zu den Analogleistungen gehören Hilfe bei Krankheit, Hilfe zur Pflege, Hilfe bei Schwangerschaft und Mutterschaft. Sie entsprechen weitgehend der Regelversorgung in der gesetzlichen Krankenversicherung (Stichwort „Regelversorgung“).

Die Leistungsberechtigten nach den §§3–6 AsylbLG haben im Vergleich zum SGB XII einen deutlich eingeschränkten Anspruch auf Gesundheitsversorgung, der sich auf die nicht aufschiebbare Akut- und Notfallversorgung konzentriert. Nach §4 Absatz 1 AsylbLG sind

zur Behandlung akuter Erkrankungen und Schmerzzustände die erforderliche ärztliche und zahnärztliche Behandlung einschließlich der Versorgung mit Arznei- und Verbandmitteln sowie sonstiger zur Genesung, zur Besserung oder zur Linderung von Krankheiten oder Krankheitsfolgen erforderlichen Leistungen zu gewähren. Zur Verhütung und Früherkennung von Krankheiten werden Schutzimpfungen entsprechend den §§47, 52 Absatz 1 Satz 1 des Zwölften Buches Sozialgesetzbuch und die medizinisch gebotenen Vorsorgeuntersuchungen erbracht. Eine Versorgung mit Zahnersatz erfolgt nur, soweit dies im Einzelfall aus medizinischen Gründen unaufschiebbar ist.

Für werdende Mütter und Wöchnerinnen sind ärztliche und pflegerische Hilfe und Betreuung, Hebammenhilfe, Arznei‑, Verband- und Heilmittel zu gewähren (§4 Abs. 2 AsylbLG). Das entspricht der normalen Grundversorgung. Daneben können nach §6 Abs. 1 AsylbLG sonstige Leistungen gewährt werden, wenn sie *im Einzelfall* zur Sicherung der Gesundheit unerlässlich ist. Personen mit einer Aufenthaltserlaubnis und besonderen Bedürfnissen,

wie beispielsweise unbegleitete Minderjährige oder Personen, die Folter, Vergewaltigung oder sonstige schwere Formen psychischer, physischer oder sexueller Gewalt erlitten haben, wird die erforderliche medizinische oder sonstige Hilfe gewährt (§6 Abs. 2 AsylbLG).

Die leistungsrechtlichen Merkmale Akutheit, Erforderlichkeit und Unaufschiebbarkeit zielen darauf, die Behandlung vor allem von chronischen Erkrankungen und ihre Versorgung mit Heil‑, Hilfs- und Pflegemitteln auf die Zeit nach dem Anerkennungsverfahren oder der Abschiebung hinauszuzögern. Die harten Restriktionen in der Leistungsgewährung werden besonders deutlich bei der (Unter-)Versorgung mit Zahnersatz. Das belegen exemplarisch sozialmedizinische Auftragsgutachten des Medizinischen Dienstes der Krankenversicherung (MDK) Rheinland-Pfalz für den Öffentlichen Gesundheitsdienst, die bei 318 vorgelegten Behandlungsplanungen im Rahmen des AsylbLG nur rund ein Viertel für die Leistungsgewährung vollumfänglich empfahlen und über die Hälfte der Anträge komplett ablehnten [13].

Die Asylbewerberleistungsstatistik wird als Sekundärstatistik in Deutschland erhoben, bei der von den zuständigen Behörden vorliegende prozessgenerierte Verwaltungsdaten aufbereitet werden. Sie wird jährlich zum Stichtag 31.12. als Vollerhebung flächendeckend durchgeführt. Die Auskunftspflicht und die Erhebungsmerkmale sind in §12 AsylbLG niedergelegt. Die Bundesstatistik berichtet die Zahl und Struktur der Leistungsberechtigten sowie die finanziellen Auswirkungen des AsylbLG. Die Daten werden in den statistischen Ämtern der Länder in einem gemeinsamen Aufbereitungsprogramm erfasst und nach Plausibilität überprüft. Qualitätsberichte informieren über die Methodik [14].

Für die vorliegende Arbeit wurden an einem Gastwissenschaftsarbeitsplatz im Forschungsdatenzentrum der Statistischen Ämter des Bundes und der Länder die beiden Scientific Use Files zu den „Empfängern von Asylbewerberregelleistungen“ und den „Empfängern von besonderen Asylbewerberleistungen“ unter Geheimhaltungsvorschriften ausgewertet. Die „Statistik der Ausgaben und Einnahmen für Asylbewerberleistungen“ steht nicht als Datensatz auf der Mikroebene, sondern in Tabellenform zur Verfügung. Die Statistiken weisen nur Personen mit bestehendem Leistungsbezug zum 31.12.2018 aus, während die Ein- und Ausgaben ganzjährig verbucht wurden. Personen, die vom BAMF die Zuerkennung der Flüchtlingseigenschaft erhalten haben oder als Asylberechtigte anerkannt sind, sind nicht mehr nach dem AsylbLG leistungsberechtigt. Die Bundesstatistiken werden alle 2 Jahre in der Fachserie 13 Reihe 7 publiziert [14]. Zu den für diese Auswertung relevanten Items zählen die Leistungsempfänger mit Geschlecht, Geburtsdatum, Staatsangehörigkeit, aufenthaltsrechtlichem Status und Wohnort. Es werden die Regelbedarfsstufen, Art und Träger der Unterkünfte sowie die gewährten Hilfearten und -formen erhoben. Bei den Empfängern von Asylbewerberregelleistungen werden darüber hinaus der Erwerbsstatus und der Leistungsbeginn registriert.

Die deskriptive Statistik und Korrelationsanalytik wurden mit der Statistiksoftware IBM SPSS Statistics 26.0 gerechnet.

## Ergebnisse

Zum Stichtag 31.12.2018 haben 423.201 Personen in Deutschland Leistungen nach dem AsylbLG erhalten. Tab. 1 führt die Leistungsempfänger von Asylbewerberregelleistungen und ausschließlich besonderen Leistungen für einen Überblick zusammen. 64,1 % der Leistungsempfänger sind Männer und 35,9 % Frauen (Tab. 1, Sp. 2). Im Durchschnitt sind die Personen 24,2 Jahre alt (SE = 0,03). Fast ein Drittel ist unter 18 Jahre alt. 53,9 % stammen aus Asien mit den wichtigsten Herkunftsländern Afghanistan, Irak, Syrien, Iran und Pakistan. 23,1 % sind Staatsangehörige von afrikanischen Ländern (Nigeria, Somalia etc.) und 20,0 % von europäischen Ländern (Russische Föderation, Türkei etc.) Die Leistungsbezieher besitzen größtenteils eine Aufenthaltsgestattung (69,7 %) oder eine Duldung (17,3 %).

**Table 1:** 

	Insgesamt		Davon mit:			
			Regelleistungen^a^ (EVAS 22221)			Ausschließlich besondere Leistungen^b^ (EVAS 22231)
	Anzahl Leistungsempfänger (Spalten 3 + 6)	Anteil in %	Insgesamt	Davon:		
				Grundleistung (§3 AsylbLG)	Leistungen in besonderen Fällen (§2 AsylbLG)	(§§2 und 3 AsylbLG)
	*(Spalte 1)*	*(Spalte 2)*	*(Spalte 3)*	*(Spalte 4)*	*(Spalte 5)*	*(Spalte 6)*
*Deutschland*	423.201	100,0	411.211	192.191	219.020	11.990
**Demografische Merkmale**						
*Geschlecht*						
Männlich	271.264	64,1	264.081	125.151	138.930	7183
Weiblich	151.937	35,9	147.130	67.040	80.090	4807
*Altersgruppen in Jahren*						
0–6	68.271	16,1	66.926	27.844	39.082	1345
7–17	63.020	14,9	61.140	24.370	36.770	1880
18–24	83.337	19,7	81.137	39.876	41.261	2200
25–29	60.650	14,3	59.010	29.818	29.192	1640
30–39	87.976	20,8	85.471	42.876	42.595	2505
40–64	55.941	13,2	53.970	25.916	28.054	1971
65 und älter	4006	0,9	3557	1491	2066	449
*Staatsangehörigkeit nach Kontinenten*						
Europa	84.769	20,0	83.397	36.634	46.763	1372
Afrika	97.572	23,1	95.792	55.967	39.825	1780
Amerika	1208	0,3	1185	841	344	23
Asien	228.263	53,9	219.827	93.300	126.527	8436
Australien, staatenlos oder ungeklärt	11.389	2,7	11.010	5449	5561	379
**Status und Leistungsbezug**						
*Aufenthaltsrechtlicher Status*						
Aufenthaltsgestattung	294.794	69,7	286.467	132.406	154.061	8327
Vollziehbar zur Ausreise verpflichtet	18.267	4,3	17.979	11.871	6108	288
Familienangehörige/-r	15.437	3,6	15.307	5400	9907	130
Geduldete/-r Ausländer/-in	73.301	17,3	70.858	33.576	37.282	2443
Einreise über einen Flughafen	369	0,1	320	97	223	49
Aufenthaltserlaubnis	3837	0,9	3340	929	2411	497
Folge- oder Zweitantrag	2385	0,6	2347	1843	504	38
Ohne Angabe^c^	14.811	3,5	14.593	6069	8524	218
*Regelbedarfsstufen*						
Alleinstehende Leistungsberechtigte	187.227	44,2	182.369	95.842	86.527	4858
Zwei erwachsene Leistungsberechtigte in gemeinsamem Haushalt	96.613	22,8	93.326	40.487	52.839	3287
Weitere erwachsene Leistungsberechtigte ohne eigenen Haushalt	8241	1,9	7616	3797	3819	625
Leistungsberechtigte Kinder und Jugendliche	131.120	31,1	127.900	52.065	75.835	3220
*Leistungsbezugsdauer*^g^						
Unter 3 Monate	–	12,8	52.488	41.665	10.823	–
3–14 Monate	–	31,4	128.335	79.579	48.756	–
15–23 Monate	–	16,1	65.936	19.178	46.758	–
24–47 Monate	–	30,5	124.898	34.494	90.404	–
48 und mehr	–	9,1	37.304	15.431	21.873	–
*Erwerbsstatus*^d,g^ *am Jahresende*						
Vollzeit	–	2,2	9134	3094	6040	–
Teilzeit	–	2,6	10.511	2252	8259	–
Arbeitsgelegenheit	–	0,3	1434	1232	202	–
Nicht erwerbstätig	–	94,9	390.132	185.613	204.519	–
**Unterbringung und Träger**						
*Art der Unterbringung*^e^						
Aufnahmeeinrichtung	44.381	10,5	44.207	33.799	10.408	174
Gemeinschaftsunterkunft	172.388	40,7	170.492	83.637	86.855	1896
Dezentrale Unterbringung	206.432	48,8	196.512	74.755	121.757	9920
*Art des Trägers*^f^						
Örtlich	320.922	75,8	309.064	123.164	185.900	11.858
Überörtlich	102.279	24,2	102.147	69.027	33.120	132
*Bundesland des Trägers*						
Baden-Württemberg	46.681	11,0	46.497	26.124	20.373	184
Bayern	65.529	15,5	64.514	37.174	27.340	1015
Berlin	25.112	5,9	25.096	8470	16.626	16
Brandenburg	15.320	3,6	15.250	6407	8843	70
Bremen	3698	0,9	3698	1236	2462	0
Hamburg	20.007	4,7	11.203	4003	7200	8804
Hessen	29.266	6,9	29.200	11.089	18.111	66
Mecklenburg-Vorpommern	5876	1,4	5853	3512	2341	23
Niedersachsen	40.136	9,5	39.806	18.291	21.515	330
Nordrhein-Westfalen	99.770	23,6	98.480	37.261	61.219	1290
Rheinland-Pfalz	16.542	3,9	16.538	8658	7880	4
Saarland	1513	0,4	1513	1043	470	0
Sachsen	21.312	5,0	21.197	10.098	11.099	115
Sachsen-Anhalt	8763	2,1	8745	7180	1565	18
Schleswig-Holstein	15.786	3,7	15.762	7161	8601	24
Thüringen	7890	1,9	7859	4484	3375	31

^a^*Datenquellen: *Forschungsdatenzentrum (FDZ) der Statistischen Ämter des Bundes und der Länder [15], eigene Berechnungen

^b^*Datenquellen: *FDZ der Statistischen Ämter des Bundes und der Länder [16], eigene Berechnungen

^c^Einschließlich Personenkreis mit Bescheinigung über die Meldung als Asylsuchender (BüMA)

^d^Hierzu zählen nur Leistungsberechtigte, die gem. §8a AsylbLG der zuständigen Behörde die Aufnahme einer unselbstständigen oder selbstständigen Erwerbstätigkeit gemeldet haben. Arbeitsgelegenheiten nach §5 AsylbLG zählen in diesem Zusammenhang nicht als Erwerbstätigkeit

^e^Als Gemeinschaftsunterkunft zählen die Unterkünfte, die von staatlicher Seite den Asylbewerbern nach der Unterbringung in einer Aufnahmeeinrichtung zur Verfügung gestellt und betreut werden. Wird eine ganze Wohnung oder der Eingang einer staatlich betriebenen Gemeinschaftsunterkunft von mehr als einem Leistungsempfängerhaushalt mit Küchen- und Sanitätsbereich genutzt, handelt es sich somit um eine Gemeinschaftsunterkunft. Dies gilt auch, wenn 2 verschiedene Haushalte in einer solchen Wohnung oder in einem Raum leben, auch wenn es nur 2 Personen sind. Besteht dagegen die Möglichkeit zur Nutzung eines eigenen Küche- und Sanitärbereichs sowie eines eigenen Wohnungseingangs und leben in dieser Wohnung nur Personen aus einem Haushalt, handelt es sich um eine dezentrale Unterbringung. Gleiches gilt, wenn der Wohnraum nicht von staatlicher Seite zur Verfügung gestellt und betreut wird und mehrere Haushalte eine Wohngemeinschaft bilden

^f^Örtliche Träger sind die für die dezentrale Durchführung des AsylbLG zuständigen Stellen auf Gemeinde- und Kreisebene. Überörtliche Träger sind höhere Kommunalbehörden sowie die Länder selbst, sofern diese für die Durchführung des AsylbLG zuständig sind

^g^Wird nicht bei Empfänger von ausschließlich besonderen Leistungen nach dem Asylbewerberleistungsgesetz erhoben

Von den Empfängern von Asylbewerberregelleistungen beziehen 44,2 % die Unterstützung mit einer Dauer bis unter 15 Monaten (Tab. 1, Sp. 2). Die Leistungsbezugsdauer beträgt im Mittel fast 2 Jahre bzw. 23,4 Monate (SE = 0,02).

10,5 % der Leistungsempfänger sind in Aufnahmeeinrichtungen untergebracht (Tab. 1, Sp. 2). 40,7 % leben in Gemeinschaftsunterkünften und 48,8 % in dezentralen Unterbringungsformen (insbesondere Einzelwohnungen). Bei knapp einem Viertel der Leistungsempfänger sind die Länder als überörtliche Träger, bei dem Rest die Gemeinden und Kreise als örtliche Träger zuständig. Die Hälfte der Leistungsempfänger verteilt sich auf die 3 größten Bundesländer Nordrhein-Westfalen, Bayern und Baden-Württemberg.

An der Regelversorgung nach §2 AsylbLG orientieren sich die analogen Leistungen entsprechend dem SGB XII, die unter den gesetzlichen Voraussetzungen den Leistungsberechtigten anstelle der Grundleistungen (nach 15 Monaten Voraufenthaltszeit) gewährt werden. Das betrifft die Hilfe zum Lebensunterhalt und die Leistungen in besonderen Fällen. Wie in Tab. 2 (Sp. 1) gezeigt, erhielten von den Leistungsempfängern 79.480 ambulante Hilfe bei Krankheit, 3099 stationäre Hilfe bei Krankheit, 324 Hilfe bei Schwangerschaft und Mutterschaft, 1585 Hilfe zur Pflege und 3774 sonstige Hilfen als Analogleistungen (§2 AsylbLG) am Jahresende nach Kapitel 5–9 SGB XII.

**Table 2:** 

	Insgesamt		Davon mit:			
			Regelleistungen^a^ (EVAS 22221)			Ausschließlich besondere Leistungen^b^ (EVAS 22231)
	Anzahl Leistungsempfänger (Spalten 3 + 6)	Inanspruchnahmequote	Insgesamt	Davon:		
				Grundleistung (§3 AsylbLG)	Leistungen in besonderen Fällen (§2 AsylbLG)	(§§2 und 3 AsylbLG)
	*(Spalte 1)*	*(Spalte 2)*	*(Spalte 3)*	*(Spalte 4)*	*(Spalte 5)*	*(Spalte 6)*
*Deutschland*	423.201	100,0	411.211	192.191	219.020	11.990
**Inanspruchnahme von Hilfen bei Krankheit, Geburt, Pflege und sonstige Hilfen**						
*Regelversorgung nach Leistung in besonderen Fällen (§2 AsylbLG) am Jahresende (5.–9. Kap. SGB XII)*						
Hilfe bei Krankheit ambulant	79.480	18,8	78.474	–	78.474	1006
Hilfe bei Krankheit stationär	3099	0,7	3035	–	3035	64
Hilfe bei Schwangerschaft und Mutterschaft	324	0,1	(Wert geheim)	–	(Wert geheim)	(Wert geheim)
Hilfe zur Pflege	1585	0,4	1562	–	1562	23
Sonstige Hilfen nach Kapitel 5–9 SGB XII	3774	0,9	3668	–	3668	106
*Minimalversorgung nach anderer Leistung (§§4–6 AsylbLG) am Jahresende*						
Leistung bei Krankheit, Schwangerschaft, Geburt in Form ambulanter Behandlung	62.151	14,7	61.610	61.610	–	541
Leistung bei Krankheit, Schwangerschaft, Geburt in Form stationärer Behandlung	2237	0,5	2215	2215	–	22
Sonstige Leistungen in Form von Sach- und/oder Geldleistungen	12.080	2,9	11.972	11.972	–	108
*Minimalversorgung nach anderer Leistung (§§4–6 AsylbLG) im Laufe des Jahres*						
Leistung bei Krankheit, Schwangerschaft, Geburt in Form ambulanter Behandlung	130.819	30,9	120.750	99.439	21.311	10.069
Leistung bei Krankheit, Schwangerschaft, Geburt in Form stationärer Behandlung	15.436	3,6	15.018	13.192	1826	418
Sonstige Leistungen in Form von Sach- und/oder Geldleistungen	42.046	9,9	41.454	41.454	–	592

^a^*Datenquellen:* Forschungsdatenzentrum (FDZ) der Statistischen Ämter des Bundes und der Länder [15], eigene Berechnungen

^b^*Datenquellen:* FDZ der Statistischen Ämter des Bundes und der Länder [16], eigene Berechnungen

Tab. 2 informiert über die medizinische Minimalversorgung gemäß §3 AsylbLG, nach dem zusätzlich zu den Grundleistungen im Bedarfsfall andere Leistungen nach §§4–6 AsylbLG gewährt werden. Dementsprechend haben 62.151 Leistungsempfänger ambulante und 2237 stationäre Leistungen bei Krankheit, Schwangerschaft oder Geburt am Jahresende (§4 AsylbLG) erhalten (Tab. 2, Sp. 1). Hinzu kommen für 12.080 Leistungsempfänger sonstige Leistungen in Form von Sach- und/oder Geldmitteln (§6 AsylbLG).

Diese Hilfearten werden für die Leistungsbezieher zum 31.12. sowohl stichtagsbezogen als auch für den gesamten Jahresverlauf registriert (Tab. 2). Wie in Spalte 2 ausgewiesen, ist der Anteil der Leistungsempfänger von ambulanten Leistungen bei Krankheit, Schwangerschaft oder Geburt dann in der Jahresbetrachtung mehr als doppelt und von stationären Behandlungsformen fast 7‑mal so hoch.

Die Minimalversorgung und die Regelversorgung können anhand der jeweils gewährten Hilfen zur Gesundheit in der Reichweite direkt verglichen werden:

Im Bereich der Minimalversorgung gemäß §3 AsylbLG haben 66.654 Personen am Jahresende 2018 eine Leistung bei Krankheit (§4 AsylbLG) und/oder eine sonstige Leistung (§6 AsylbLG) in Anspruch genommen. Bezogen auf die leistungsberechtigte Grundgesamtheit von 229.818 Personen (Tab. 3, Sp. 10–11) kann eine Inanspruchnahmequote von 29,0 % am Jahresende bestimmt werden. Dem steht die gesundheitsbezogene Regelversorgung (§2 AsylbLG) analog dem SGB XII gegenüber. Hier wurden am Jahresende 82.579 Leistungsempfängern ambulante oder stationäre Hilfe bei Krankheit gewährt. Das entspricht bei einer leistungsberechtigten Grundgesamtheit von 193.383 Personen (Tab. 3, Sp. 8–9) einer wesentlich höheren Inanspruchnahme von 42,7 %. Der direkte Vergleich zeigt im Ergebnis, dass von der wohlfahrtsstaatlich großzügigeren Regelung ein höherer Prozentsatz unter den Leistungsberechtigten profitiert hat (42,7 % vs. 29,0 %).

**Table 3:** 

	Insgesamt (*N* = 423.201)							Regelversorgung am Jahresende (analog SGB XII; *N* = 193.383)		Minimalversorgung am Jahresende (*N* = 229.818)	
	Keine Krankenbehandlung		Ambulante Behandlung^a^ (Spalten 8 + 10)		Stationäre Behandlung (Spalten 9 + 11)		*p*-Wert	Ambulante Hilfe bei Krankheit in besonderen Fällen (§2 AsylbLG)	Stationäre Hilfe bei Krankheit in besonderen Fällen (§2 AsylbLG)	Ambulante Leistung bei Krankheit, Schwangerschaft, Geburt (§§4 AsylbLG)	Stationäre Leistung bei Krankheit, Schwangerschaft, Geburt (§§4 6 AsylbLG)
	Anzahl	Prävalenz	Anzahl	Prävalenz	Anzahl	Prävalenz		Anzahl	Anzahl	Anzahl	Anzahl
	*(Spalte 1)*	*(Spalte 2)*	*(Spalte 3)*	*(Spalte 4)*	*(Spalte 5)*	*(Spalte 6)*	*(Spalte 7)*	*(Spalte 8)*	*(Spalte 9)*	*(Spalte 10)*	*(Spalte 11)*
*Deutschland*	276.235	65,3 %	141.630	33,5 %	5336	1,3 %	–	79.480	3099	62.151	2237
**Demografische Merkmale**											
*Geschlecht*											
Männlich	179.510	66,2 %	88.635	32,7 %	3119	1,1 %	*p* < 0,001	49.194	1813	39.442	1306
Weiblich	96.725	63,7 %	52.995	34,9 %	2217	1,5 %		30.286	1286	22.709	931
*Altersgruppen in Jahren*											
0–6	44.517	65,2 %	22.772	33,4 %	982	1,4 %	*p* < 0,001	13.986	633	8786	349
7–17	40.010	63,5 %	22.285	35,4 %	725	1,2 %		13.995	439	8290	286
18–24	56.630	68,0 %	25.722	30,9 %	985	1,2 %		13.671	517	12.051	468
25–29	40.425	66,7 %	19.493	32,1 %	732	1,2 %		10.124	402	9369	330
30–39	57.138	64,9 %	29.774	33,8 %	1064	1,2 %		15.674	590	14.101	474
40–64	35.164	62,9 %	20.018	35,8 %	759	1,4 %		11.024	456	8994	303
65 und älter	2351	58,7 %	1566	39,1 %	89	2,2 %		1006	62	560	27
*Staatsangehörigkeit nach Kontinenten*											
Europa	52.594	62,0 %	30.937	36,5 %	1238	1,5 %	*p* < 0,001	18.730	826	12.207	412
Afrika	65.389	67,0 %	31.061	31,8 %	1122	1,1 %		12.643	490	18.418	632
Amerika	767	63,5 %	430	35,6 %	11	0,9 %		163	3	267	8
Asien	149.227	65,4 %	76.151	33,4 %	2885	1,3 %		46.336	1739	29.816	1146
Australien, staatenlos oder ungeklärt	8258	72,5 %	3051	26,8 %	80	0,7 %		1608	41	1443	39
**Status und Leistungsbezug**											
*Aufenthaltsrechtlicher Status*											
Aufenthaltsgestattung	195.272	66,2 %	95.704	32,5 %	3818	1,3 %	*p* < 0,001	54.606	2228	41.098	1590
Vollziehbar zur Ausreise verpflichtet	10.210	55,9 %	7889	43,2 %	168	0,9 %		2946	44	4943	124
Familienangehörige/-r	10.696	69,3 %	4590	29,7 %	151	1,0 %		2795	122	1795	29
Geduldete/-r Ausländer/-in	46.755	63,8 %	25.519	34,8 %	1027	1,4 %		13.552	609	11.967	418
Aufenthaltserlaubnis	2367	61,7 %	1424	37,1 %	46	1,2 %		993	26	432	20
Folge- oder Zweitantrag	1854	77,7 %	509	21,3 %	22	0,9 %		185	12	324	10
Ohne Angabe^c^ oder Einreise über Flughafen	9081	59,8 %	5995	39,5 %	104	0,7 %		4403	58	1592	46
*Regelbedarfsstufen*											
Alleinstehende Leistungsberechtigte	126.058	67,3 %	59.097	31,6 %	2072	1,1 %	*p* < 0,001	29.565	1080	29.533	992
Zwei erwachsene Leistungsberechtigte in gemeinsamen Haushalt	60.207	62,3 %	35.030	36,3 %	1376	1,4 %		20.583	870	14.447	506
Weitere erwachsene Leistungsberechtigte ohne eigenen Haushalt	5583	67,7 %	2471	30,0 %	187	2,3 %		1350	81	1121	106
Leistungsberechtigte Kinder und Jugendliche	84.387	64,4 %	45.032	34,3 %	1701	1,3 %		27.982	1068	17.050	633
**Unterbringung und Träger**											
*Art der Unterbringung*^e^											
Aufnahmeeinrichtung	35.181	79,3 %	8867	20,0 %	333	0,8 %	*p* < 0,001	3737	98	5130	235
Gemeinschaftsunterkunft	98.331	57,0 %	72.865	42,3 %	1192	0,7 %		35.801	765	37.064	427
Dezentrale Unterbringung	142.723	69,1 %	59.898	29,0 %	3811	1,8 %		39.942	2236	19.957	1575
*Art des Trägers*^f^											
Örtlich	190.045	59,2 %	126.543	39,4 %	4334	1,4 %	*p* < 0,001	74.333	2594	52.211	1740
Überörtlich	86.190	84,3 %	15.087	14,8 %	1002	1,0 %		5147	505	9940	497
*Bundesland des Trägers*											
Baden-Württemberg	33.689	72,2 %	12.949	27,7 %	43	0,1 %	*p* < 0,001	2849	13	10.100	30
Bayern^g^	51.931	79,2 %	12.987	19,8 %	611	0,9 %		7171	396	5816	215
Berlin^b^	(25.055)	(99,8 %)	(24)	(0,1 %)	(33)	(0,1 %)		(17)	(23)	(7)	(10)
Brandenburg^g^	(9098)	(59,4 %)	(6174)	(40,3 %)	(48)	(0,3 %)		(3011)	(10)	(3163)	(38)
Bremen^g^	1262	34,1 %	2389	64,6 %	47	1,3 %		1353	47	1036	0
Hamburg	17.648	88,2 %	2120	10,6 %	239	1,2 %		2079	234	41	5
Hessen	16.352	55,9 %	12.726	43,5 %	188	0,6 %		7505	175	5221	13
Mecklenburg-Vorpommern	3215	54,7 %	2635	44,8 %	26	0,4 %		990	14	1645	12
Niedersachsen	25.918	64,6 %	13.008	32,4 %	1210	3,0 %		5710	326	7298	884
Nordrhein-Westfalen	53.415	53,5 %	45.435	45,5 %	920	0,9 %		29.737	710	15.699	210
Rheinland-Pfalz^g^	9136	55,2 %	7245	43,8 %	161	1,0 %		5455	151	1790	10
Saarland	1237	81,8 %	276	18,2 %	0	0 %		255	0	21	0
Sachsen	9642	45,2 %	11.629	54,6 %	41	0,2 %		10.294	31	1335	10
Sachsen-Anhalt	2078	23,7 %	6444	73,5 %	241	2,8 %		282	222	6162	19
Schleswig-Holstein	11.963	75,8 %	2733	17,3 %	1090	6,9 %		1635	485	1098	605
Thüringen^g^	4596	58,3 %	2856	36,2 %	438	5,6 %		1137	262	1719	176

Quelle: Forschungsdatenzentrum (FDZ) der Statistischen Ämter des Bundes und der Länder [15, 16], eigene Berechnungen

^a^Bei einer Mehrfachnennung für 1 Leistungsempfänger von ambulanten und stationären Leistungen am Jahresende wird nur die stationäre Leistung ausgewiesen

^b^Über das Amt für Statistik Berlin-Brandenburg wurde mitgeteilt, dass die Asylbewerberleistungsstatistiken bezüglich der Leistungen zu Hilfen bei Krankheit nicht aussagekräftig sind. Die Ursache sind abrechnungstechnische Gründe. Wenn die Leistungsempfänger in Berlin und Brandenburg wegen der Untererfassung der Krankheitshilfen nicht berücksichtigt werden, liegt bei den übrigen 14 Bundesländern der Anteil aller Leistungsempfänger mit ambulanter Behandlung insgesamt bei 35,4 % und mit stationärer Behandlung bei 1,4 % am Jahresende 2018

^c–f^Siehe Anmerkungen Tab. 1

^g^Nach einer Abfrage des Statistischen Bundesamts bei allen Bundesländern im Februar 2020 (im Zuge dieses Forschungsprojekts) ist nach den Antworten von einer statistischen Untererfassung der ambulanten und stationären Hilfen bei Krankheit nach dem Asylbewerberleistungsgesetz aufgrund der jeweiligen regionalen Fachverfahren auszugehen

Gleichzeitig demonstriert die Gegenüberstellung in Tab. 4 (Spalten 8 vs. 5), dass die Regelversorgung mit analogen gesundheitsrelevanten Hilfen in besonderen Fällen (§2 AsylbLG analog SGB XII) nicht nur mehr leistungsberechtigte Menschen in Deutschland erreicht, sondern bei den Bruttoausgaben insgesamt weniger Kosten als die bürokratieaufwendige Minimalversorgung nach §3 AsylbLG verursacht hat.

**Table 4:** 

	Leistungsempfänger insgesamt (kalkulatorischer Jahresdurchschnitt)	Darunter: Minimalversorgung im Laufe des Jahres (IL)^a^			Bruttoausgaben^b^ (EVAS 22211-0014) Darunter: nach Hilfearten im Laufe des Jahres (IL)			
	(Bestand an Leistungsempfängern zum 31.12.2018 plus halbe Differenz zum Bestand am 31.12.2017)	Ambulante und stationäre Leistung bei Krankheit, Schwangerschaft, Geburt (§4 AsylbLG)	Sonstige Leistungen (§6 AsylbLG); Geld- und/oder Sachleistung	Ambulante oder stationäre Leistung oder Sachleistung (§§4 und 6 AsylbLG)	Minimalversorgung			Regelversorgung
					Insgesamt (§4, 6 AsylbLG)	Davon: Leistung bei Krankheit, Schwangerschaft und Geburt (§4 AsylbLG)	Davon: sonstige Leistungen (§6 AsylbLG)	Leistung in besonderen Fällen (§2 AsylbLG: 5.–9. Kap. SGB XII)
	Anzahl IL	Anzahl IL	Anzahl IL	Anzahl IL	In Tsd. EUR	In Tsd. EUR	In Tsd. EUR	In Tsd. EUR
	*(Spalte 1)*	*(Spalte 2)*	*(Spalte 3)*	*(Spalte 4)*	*(Spalte 5)*	*(Spalte 6)*	*(Spalte 7)*	*(Spalte 8)*
*Deutschland*	446.951	133.115	42.046	144.269	536.724	492.472	44.252	430.919
*Bundesland des Trägers*								
Baden-Württemberg	52.021	21.121	6942	22.682	61.646	56.049	5597	35.165
Bayern^c^	66.970	14.466	7236	17.149	85.869	78.809	7060	77.307
Berlin^d^	25.366	(47)	(632)	(666)	28.573	26.562	2011	37.085
Brandenburg^c^	15.331	5919	2460	6410	20.682	18.504	2178	17.432
Bremen^c^	3820	1452	204	1484	11.820	10.970	850	1215
Hamburg	16.018	14.407	494	14.721	20.135	18.596	1539	17.604
Hessen	31.447	8273	3549	8987	28.968	25.493	3475	27.597
Mecklenburg-Vorpommern	6113	3351	848	3405	8997	8354	643	4753
Niedersachsen	42.428	12.993	5101	14.247	43.808	36.077	7731	46.244
Nordrhein-Westfalen	111.590	20.809	3671	21.643	121.213	115.837	5376	107.436
Rheinland-Pfalz^c^	17.857	7700	4843	8188	25.661	22.793	2868	7673
Saarland	1535	58	907	954	2953	2662	291	687
Sachsen^c^	22.198	9623	1259	9759	25.522	23.792	1730	24.692
Sachsen-Anhalt	9475	6996	1104	7105	13.534	12.687	847	4433
Schleswig-Holstein	16.258	3108	1885	3592	35.562	34.204	1358	21.136
Thüringen^c, e^	8524	2792	911	3277	(1781)	(1083)	(698)	(459)

^a^Datenquelle: Forschungsdatenzentrum (FDZ) der Statistischen Ämter des Bundes und der Länder [15, 16], eigene Berechnungen

^b^Datenquelle: Asylbewerberleistungsstatistik „Statistik der Ausgaben und Einnahmen für Asylbewerberleistungen“ [17]

^c^Spalten 2–4: Nach einer Abfrage des Statistischen Bundesamts bei allen Bundesländern im Februar 2020 (im Zuge dieses Forschungsprojekts) ist von einer statistischen Untererfassung der ambulanten und stationären Hilfen bei Krankheit nach dem Asylbewerberleistungsgesetz aufgrund der jeweiligen regionalen Fachverfahren auszugehen

^d^Spalten 2–4: Über das Amt für Statistik Berlin-Brandenburg wurde mitgeteilt, dass die Asylbewerberleistungsstatistiken bezüglich der Leistungen zu Hilfen bei Krankheit nicht aussagekräftig sind. Die Ursache sind abrechnungstechnische Gründe

^e^Spalten 6–9: Das Bundesland Thüringen wird in der Abb. 2 nicht separat ausgewiesen, weil es nach einer Länderabfrage des Statistischen Bundesamts im Februar 2020 (im Zuge dieses Forschungsprojekts) die Gesundheitsausgaben aufgrund des regionalen Fachverfahrens nicht valide statistisch erfasst

Es wird außerdem davon ausgegangen, dass verwaltungsmäßige Schwierigkeiten bei Jahresabgrenzungen in den Rechnungsstellungen in allen Bundesländern bestehen

Tab. 3 kombiniert die beiden Hilfearten bei Krankheit nach §§2 und 3 AsylbLG. Für alle Leistungsempfänger werden gemeinsame Prävalenzraten sowohl der ambulanten als auch der stationären Krankenversorgung berechnet. Zum Stichtag 31.12.2018 befanden sich demnach 33,5 % aller Leistungsempfänger in ambulanter Behandlung und 1,3 % in stationärer Behandlung. Die Prävalenzraten variieren innerhalb der soziodemografischen Gruppen in Tab. 3 in statistisch signifikanter Weise und sind bei den Frauen, Älteren und Angehörigen europäischer Staaten leicht erhöht. Auffällig hoch ist der Anteil mit ambulanter Krankenversorgung in der Personengruppe, die vollziehbar zur Ausreise verpflichtet ist.

Zum Schutz der Bevölkerung werden nach §62 Asylgesetz (AsylG) alle Ausländer, die in einer Aufnahmeeinrichtung oder Gemeinschaftsunterkunft zu wohnen haben, verpflichtet, eine ärztliche Untersuchung auf übertragbare Krankheiten einschließlich einer Röntgenaufnahme der Atmungsorgane zu dulden. Der Öffentliche Gesundheitsdienst ist angehalten, den Impfstatus von Asylsuchenden systematisch zu kontrollieren und mit freiwilligen Angeboten auf die Schließung von Impflücken gemäß den Empfehlungen der Ständigen Impfkommission (STIKO) frühzeitig hinzuwirken [18]. Speziell für die Aufnahmeeinrichtungen, die in der Zuständigkeit der Länder liegen, zeigt sich hier (entgegen den Erwartungen) kein Sondereffekt (siehe Tab. 3). In den Aufnahmeeinrichtungen sowie generell bei überörtlichen Trägern gibt es eine weit unterdurchschnittliche Gewährung von ambulanten oder stationären Leistungen bei Krankheit.

**Figure 1:**
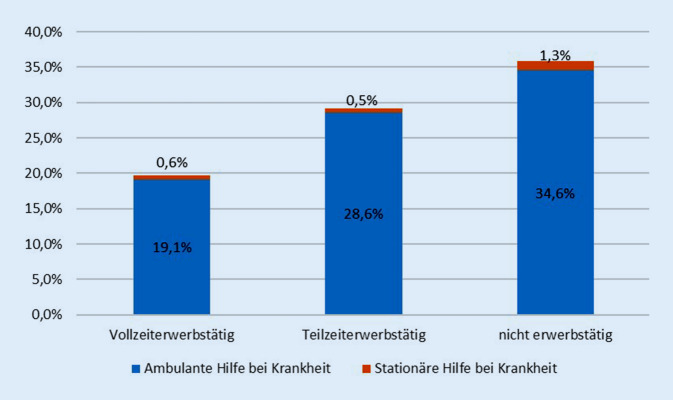

4,8 % der Empfänger von Asylbewerberregelleistungen sind erwerbstätig (davon 89,3 % Männer). Abb. 1 veranschaulicht eine enge Assoziation zwischen Krankheitshilfen und der Arbeitsmarktintegration. Erwartungsgemäß ist der Anteil mit ambulanten oder stationären Leistungen bei Krankheit unter den Empfängern von Asylbewerberregelleistungen in Erwerbstätigkeit signifikant geringer als in Nichterwerbstätigkeit am Jahresende (Chi-Quadrat = 1237,4; df = 4; *p* < 0,001).

Am Jahresende 2018 ist bei den verschiedenen Bundesländern eine außerordentlich große Bandbreite hinsichtlich der Gewährung von Leistungen bei Krankheit zu beobachten. Sie reicht von 11,8 % der Leistungsempfänger in Hamburg bis in der Spitze zu 76,3 % in Sachsen-Anhalt (Tab. 3, Sp. 4 + 6).

**Figure 2:**
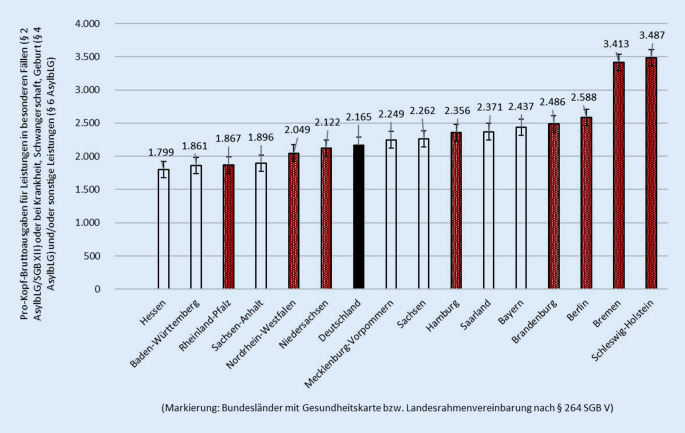

Abb. 2 basiert auf den amtlichen Angaben zu den Bruttoausgaben für den gesamten Jahresverlauf (Tab. 4, Sp. 5–8). Nachdem die Leistungsempfänger nur punktuell zum 31.12. erhoben werden, wird als Bezugsgröße der Jahresdurchschnitt für eine Näherung geschätzt. Der Vorjahresbestand zum 31.12.2017 war nach der Statistik in allen Bundesländern höher und nahm jeweils prozentual verschieden bis zum 31.12.2018 ab. Die Differenz zwischen den Beständen am Beginn und Ende 2018 wird daher mit der Hälfte für einen kalkulatorischen Jahresdurchschnitt auf der Bundesländerebene berücksichtigt (Tab. 4, Sp. 1).

Nach dieser groben Kalkulation liegen die Bruttoausgaben für Leistungen in besonderen Fällen (§2 AsylbLG) oder bei Krankheit, Schwangerschaft, Geburt (§4 AsylbLG) und/oder sonstige Leistungen (§6 AsylbLG) pro Kopf im Jahresdurchschnitt bei insgesamt 2165 € in Deutschland. Abb. 2 illustriert, dass die Länder Schleswig-Holstein und Bremen auf dieser Berechnungsgrundlage mit weitem Abstand die höchsten Pro-Kopf-Bruttoausgaben haben (3487 € und 3413 €), während am anderen Pol in Hessen lediglich etwas über der Hälfte verausgabt wurde.

Die 4 (kleinen) Bundesländer mit den höchsten Pro-Kopf-Bruttoausgaben für Gesundheitsleistungen haben eine Landesrahmenvereinbarung nach § 264 SGB V für die Gesundheitskarte abgeschlossen. Die Bundesländer mit Gesundheitskarte weisen eine signifikant höhere Inanspruchnahmequote von stationären Leistungen am Jahresende als die übrigen Bundesländer mit 1,7 % vs. 0,6 % auf (Chi-Quadrat = 968,2; df = 2; *p* < 0,001). Bei der ambulanten Versorgung gibt es dagegen kaum Unterschiede.

Eine Heterogenität im Leistungsgeschehen zeigt sich nicht nur zwischen den Bundesländern, sondern auch zwischen den Kreisen, die aber aus Geheimhaltungsgründen nicht dargestellt werden kann. So wurden in 17 Kreisen für die gemeldeten Leistungsempfänger am Jahresende überhaupt keine ambulanten oder stationären Behandlungsfälle angegeben.

## Diskussion und Fazit

Die Asylbewerberstatistik ist als jährliche Vollerhebung am Jahresende angelegt und das AsylbLG verpflichtet, über die Erhebungsmerkmale Auskunft zu geben. Dabei sind methodische Limitationen zu beachten. Da der objektive medizinische Versorgungsbedarf (z. B. durch Screenings) bei asylsuchenden Menschen nicht statistisch erhoben wird, bestehen Limitationen bei der Interpretation der unterschiedlichen Inanspruchnahmen von Krankheitshilfen zwischen den Gruppen oder Regionen. Ein Bias entsteht, wenn die Berichtsstellen einzelne Fälle bzw. Hilfen nicht oder nachträglich erfassen. Nach der berichteten Abfrage des Statistischen Bundesamts im Februar 2020 ist von einer statistischen Untererfassung der ambulanten und stationären Hilfen bei Krankheit nach dem Asylbewerberleistungsgesetz in verschiedenen Bundesländern auszugehen (vgl. Anmerkungen bei Tab. 3–4 und Abb. 1–2). Der Vergleich zwischen den Bundesländern offenbart eine große Heterogenität im ambulanten und stationären Leistungsgeschehen bei Krankheit und muss daher besonders vorsichtig interpretiert werden. In den bevölkerungsstarken Bundesländern wie in Nordrhein-Westfalen oder Baden-Württemberg scheint die Erfassung nach den Rückmeldungen wesentlich besser zu funktionieren, was auch weniger statistische Unsicherheiten für die Bundesdurchschnittswerte bedeutet. Eine zeitliche Jahresabgrenzung ist in der offiziellen Statistik und in der Software jedoch für die Ausgabenrechnung generell nicht vorgesehen.

Die Leistungsempfänger werden nur im Sinne einer Bestandsstatistik zum 31.12. erfasst. Für weitergehende Analysen fehlen Zugangs- und Abgangsstatistiken im Jahreszeitraum. Die Ein- und Ausgabenrechnung bezieht sich dagegen auf alle Verbuchungen im Jahresverlauf. Methodisch lassen sich daher bei den Verknüpfungen zwischen den Bruttoausgaben und den Leistungsempfängern nach Hilfearten nur Näherungswerte eruieren. Wünschenswert wäre in der Zukunft die Zuordnung der Bruttoausgaben auf der Personenebene und bei den Krankheitshilfen mindestens die Registrierung der Hauptdiagnosegruppe. Hier besteht weiterer Forschungsbedarf.

In der wissenschaftlichen Perspektive liegen auf der einen Seite die Probleme bei den Asylbewerberleistungsstatistiken als Datenquelle für die Gesundheitsversorgung in der Erhebungskonzeption und in der Untererfassung in einigen Bundesländern. Die offiziellen Fachserien und Qualitätsberichte negieren bis dato die Einflüsse auf die Qualität der Gesundheitsdaten (vgl. [14]). Der Zweck dieser Erhebungen als Bundesstatistik ist nach §12 AsylbLG, die „Auswirkungen dieses Gesetzes und zu seiner Fortentwicklung zu beurteilen“. Für den Gesundheitsbereich müsste dafür die Datenbasis weiterentwickelt werden. Eine große Chance läge im Aufbau einer analogen Gesundheitsberichterstattung über die Routinen in der gesetzlichen Krankenversicherung für die Bundesländer, die die Gesundheitskarte für Asylsuchende bereits eingeführt haben.

Auf der anderen Seite haben das Erhebungsinstrument und die Forschungsdatensätze große methodische Vorzüge. Alleinstellungsmerkmale sind die flächendeckende Vollerhebung der Leistungsempfänger nach dem AsylbLG mit gesetzlicher Auskunftspflicht und die jährliche Wiederholung identischer Items. Mit der vorliegenden Studie werden erstmals die Forschungsdatensätze mit den gewährten ambulanten und stationären Leistungen bei Krankheit nach dem AsylbLG umfassend wissenschaftlich analysiert. Bisher werden ambulante und stationäre Krankheitshilfen in den Bundesstatistiken nicht separat ausgewiesen. Die Auswertungen geben nicht nur Einblick in die unterschiedlichen Inanspruchnahmen von Krankheitshilfen bei den Leistungsberechtigten nach soziodemografischen Gruppen, sondern auch nach Status, Leistungsbezug und Unterbringungsart. So werden in den Aufnahmeeinrichtungen und im Zuständigkeitsbereich von überörtlichen Trägern relativ wenig Krankheitshilfen bei den Leistungsempfängern am Jahresende 2018 registriert. Das ist bemerkenswert, weil mit anderen Datenquellen festgestellt wurde, dass Flüchtlinge in derartigen Einrichtungen häufiger unter psychischen Erkrankungen als in dezentralen Wohnformen leiden [8]. Die großen Fallzahlen lassen auch Analysen zu kleinen Statusgruppen wie die vollziehbar zur Ausreise verpflichteten Leistungsempfänger (mit hoher Inanspruchnahme von Krankheitshilfen) zu. Die Auswertung zeigt eine höhere Inanspruchnahme von Krankheitshilfen bei den nichterwerbstätigen Asylsuchenden im Vergleich zu Teilzeit- und Vollzeitbeschäftigten. Das bestätigt eine frühere Befragung bei Flüchtlingen, die höhere psychische Disstresswerte in Erwerbslosigkeit ergab [19]. Am größten ist die Heterogenität bei der Gewährung von Leistungen bei Krankheiten allerdings zwischen den Bundesländern. Das deckt sich mit den von Razum et al. [10] sowie von Wächter-Raquet [5] beschriebenen bundesweit uneinheitlichen Gesundheitsversorgungsstrukturen bei Asylsuchenden.

Die Prävalenzraten speziell in der stationären Krankenversorgung variieren sowohl zwischen der Minimal- und Regelversorgung nach §§2 und 3 AsylbLG als auch zwischen den Bundesländern mit und ohne Gesundheitskarte beträchtlich (Tab. 3). Nachdem die Einweisung in ein Krankenhaus durch den behandelnden Arzt oder die Krankenhausambulanz erfolgt, sind diese regionalen Befunde ein Indiz für eine medizinisch nicht bedarfsgerechte Versorgung von asylsuchenden Menschen. Die Indizien legen nahe, dass die Gesundheitskarte den Zugang in die Krankenhausversorgung erleichtert, was auch die höheren Pro-Kopf-Ausgaben in Bremen und Schleswig-Holstein erklären dürfte (Abb. 2) und konform zu Länderberichten von Wächter-Raquet [5] ist. Die frühere Behandlung von schwerwiegenden Erkrankungen könnte den Heilungsverlauf in der Prognose positiv beeinflussen und das Risiko für spätere Folgekosten aufgrund von Verchronifizierung etc. absenken. Eine offene Forschungsfrage ist, welche Einflussnahme Zuzahlungen (z. B. Krankenhaustagegeld) in Bezug auf die Inanspruchnahmen in dieser Zielgruppe ausüben.

Aus diesen Diskussionssträngen lassen sich zwei Hauptempfehlungen ableiten:

1. Die Anwendung des §2 AsylbLG schon bei einer Voraufenthaltszeit ab 3 Monaten, um frühzeitiger und bei mehr Leistungsberechtigten die Leistungen analog zu Kap. 5–9 SGB XII gewähren zu können. Die obigen Ergebnisse deuten an, dass diese Variante nicht nur bedarfsgerechter, sondern insgesamt kosteneffizienter für Deutschland ist. Das steht auch in Übereinstimmung mit Bozorgmehr und Razum [3].
2. Die flächendeckende Einführung der Gesundheitskarte in Deutschland nach einheitlichen Standards.

Die beiden Maßnahmen würden nicht nur dem Anspruch der Universalität der Menschenrechte und dem ungedeckten medizinischen Bedarf der Asylsuchenden gerecht werden, sondern auch dazu beitragen, die Gesundheitsversorgung für Asylsuchende zu vereinheitlichen und die im AsylbLG schon angelegte Zweiklassenmedizin in Deutschland zu überwinden. Darüber hinaus würden sie zur Entbürokratisierung im Gesundheitswesen und Entlastung des Öffentlichen Gesundheitsdienstes beitragen. Auf den durch die Gesundheitskarte generierten GKV-Routinedaten könnten dann auch die Gesundheitsforschung und die Bundesstatistik für das AsylbLG einheitlich und flächendeckend aufbauen. Die aktuelle Gesundheitskrise durch die SARS-CoV-2-19-Pandemie verschärft die Situation von Flüchtlingen gerade in den Aufnahme- und Gemeinschaftsunterkünften und erfordert ein schnelles Handeln [20].
